# Hyperhomocysteinemia dysregulates plasma levels of polyunsaturated fatty acids-derived eicosanoids

**DOI:** 10.53388/2022-0106-103

**Published:** 2022-04

**Authors:** Mohamed Al-Shabrawey, Ahmed Elmarakby, Yara Samra, Mohamed Moustafa, Stephen W. Looney, Krishna Rao Maddipati, Amany Tawfik

**Affiliations:** 1 Department of Foundational Medical Studies and Eye Research Center, Oakland University William Beaumont School of Medicine, Rochester, Michigan, USA.; 2 Eye Research Institute, Oakland University, Rochester, Michigan, USA.; 3 Department of Oral Biology and Diagnostic Sciences, Dental College of Georgia, Augusta University, Augusta, Georgia, USA.; 4 Departments of Pharmacology & Toxicology, Faculty of Pharmacy, Mansoura University, Mansoura, Egypt.; 5 Department of Biochemistry, Faculty of Pharmacy, Mansoura University, Egypt.; 6 Department of Population Health Sciences, Medical College of Georgia, Augusta University, Augusta, Georgia, USA.; 7 Bioactive Lipids Research Program, Department of Pathology, Wayne State University, Michigan, USA.

**Keywords:** homocysteine, cystathionine β-synthase, eicosanoids, cycloxygenase, lipoxygenase, cytochrome-P450

## Abstract

Hyperhomocysteinemia (HHcy) contributes to the incidence of many cardiovascular diseases (CVD). Our group have previously established crucial roles of eicosanoids and homocysteine in the incidence of vascular injury in diabetic retinopathy and renal injury. Using cystathionine-β-synthase heterozygous mice (cβs^+/−^) as a model of HHcy, the current study was designed to determine the impact of homocysteine on circulating levels of lipid mediators derived from polyunsaturated fatty acids (PUFA). Plasma samples were isolated from wild-type (WT) and cβs^+/−^ mice for the assessment of eicosanoids levels using LC/MS. Plasma 12/15-lipoxygenase (12/15-LOX) activity significantly decreased in cβs^+/−^ vs. WT control mice. LOX-derived metabolites from both omega-3 and omega-6 PUFA were also reduced in cβs^+/−^ mice compared to WT control (*P* < 0.05). Contrary to LOX metabolites, cytochrome P450 (CYP) metabolites from omega-3 and omega-6 PUFA were significantly elevated in cβs^+/−^ mice compared to WT control. Epoxyeicosatrienoic acids (EETs) are epoxides derived from arachidonic acid (AA) metabolism by CYP with anti-inflammatory properties and are known to limit vascular injury, however their physiological role is limited by their rapid degradation by soluble epoxide hydrolase (sEH) to their corresponding diols (DiHETrEs). In cβs^+/−^ mice, a significant decrease in the plasma EETs bioavailability was obvious as evident by the decrease in EETs/ DiHETrEs ratio relative to WT control mice. Cyclooxygenase (COX) metabolites were also significantly decreased in cβs^+/−^ vs. WT control mice. These data suggest that HHcy impacts eicosanoids metabolism through decreasing LOX and COX metabolic activities while increasing CYP metabolic activity. The increase in AA metabolism by CYP was also associated with increase in sEH activity and decrease in EETs bioavailability. Dysregulation of eicosanoids metabolism could be a contributing factor to the incidence and progression of HHcy-induced CVD.

## Introduction

Homocysteine is a naturally occurring amino acid found in blood plasma. Homocysteine is produced as an intermediate metabolite of methionine metabolism through two pathways [1, 2]. The first pathway is irreversible trans-sulphuration of cystathionine by cystathionine β-synthase and the second is the remethylation to methionine by methionine synthase [1, 2]. Although homocysteine is essential for normal cardiovascular function, abnormal increase in plasma levels of homocysteine has been shown to be a contributing factor to cardiovascular diseases (CVD) such as atherosclerosis and thrombosis and to Alzheimer disease (AD) [3, 4]. Elevated levels of homocysteine resulting from disorder in methionine metabolism lead to hyperhomocysteinemia (HHcy) and homocysteinuria. HHcy or homocysteinuria is linked to a genetic defect in the cystathionine β-synthase (cβs) gene, vitamin B deficiency and deficiency of other enzymes involved in methionine metabolism and could lead to mental and physical retardation, occlusive CVD, atherosclerosis and coronary artery disease [5-7].

The mechanisms by which homocysteine contributes to diseases such as cardiovascular and neurological dysfunction are not fully understood. However, oxidative stress, depletion of nitric oxide (NO) and epigenetic modification have been suggested as potential mechanisms that lead to vascular inflammation [8-10]. Endothelial dysfunction as a result of elevation in oxidative stress and decreased NO bioavailability is known to play a major role in cardiovascular complications seen in HHcy [11-14].

Eicosanoids are bioactive lipids derived from polyunsaturated fatty acids (PUFA), such as arachidonic acid (AA), through 3 main enzymatic pathways including the cyclooxygenase (COX), cytochrome-P450 (CYP) and lipoxygenases (LOX) that convert omega-3 and omega-6 PUFA to bioactive lipids with divergent biological functions [13, 14]. Growing evidence suggests that dysregulation of eicosanoid levels is a contributing factor to the pathogenesis of CVD complications of HHcy [15, 16]. First, COX enzymes known as COX1 (constitutive) and COX2 (inducible), metabolize AA to produce several prostaglandins (PGs), prostacyclin (PGI2) and thromboxane A2 (TXA2). These metabolites are involved in regulation of immune response, pain sensation, vascular tone, thrombus formation and platelet aggregation [17]. Second, LOX families, includes 5-LOX, 12-LOX and 15-LOX. The primary metabolites of 5-LOX are 5-hydroxyicosatetraenoic acid (5-HETE), and various leukotrienes (LTs) that contribute to inflammation, vascular hyperpermeability and bronchoconstriction [18, 19]. 12- and 15-LOX metabolize AA to 12- and 15-HETEs, respectively where these metabolites are known to increase vascular inflammatory and permeability [20, 21]. However, the metabolites of 12/15-LOX from omega-3 PUFA such as lipoxin, resolvins and neuroprotection-D2 exert anti-inflammatory properties [22-24]. The CYP450 pathway, includes epoxygenase and ω-hydroxylase, which generate epoxyeicosatrienoic acids (EETs) and 20-hydroxy-eicosatetraenoic acids (20-HETE), respectively. These metabolites also play a role in the regulation of vascular injury [25, 26]. The 20-HETE is a pro-inflammatory metabolite with potent vasoconstrictor properties that promotes endothelial dysfunction and progression of CVD [27], whereas EETs are endothelial derived hyperpolarizing factors with anti-inflammatory properties and beneficial effects in CVD28,29. However, EETs are rapidly metabolized by the soluble epoxide hydrolase enzyme (sEH) to produce less active dihydroxyeicosatrienoic acids (DiHETrEs) with limited physiological benefits [28, 29].

Linoleic acid (LA) is a precursor of the AA, which gives rise to several pro-inflammatory eicosanoids. Dietary supplementation of LA lowers risk of CVD events such as inflammation and hypertension [30, 31]. LA can be metabolized by LOX to generate 9- and 13-HODEs, and by CYP to generate epoxyoctadecanoic acids (EpOMEs) which are substrates for sEH to produce dihydroxyoctadecanoic acids metabolites (DiHOMEs) [32]. Because HHcy plays a crucial role in CVD and studies also suggest a role of eicosanoids in the pathogenesis of CVD complications of HHcy, the current study was designed to determine the change in plasma eicosanoids levels in cβs deficient mice (cβs^+/−^) as a model of HHcy relative to their control wild-type (WT) counterpart.

## Material and Methods

### Animals

Generation of mice deficient in cβs has been previously established [33]. Breeding pairs of cβs^+/−^ mice (B6.129P2-cβs^tm1Unc^/J; Jackson Laboratories, Bar Harbor, ME) were used to establish our colony of cβs^+/+^, cβs^+/−^, and cβs^−/−^ mice. For lipid analysis, male and female wild-type (WT, cβs^+/+^) and heterozygous (cβs^+/−^) mice were used at ages ranging from 4-6 months as cβs^−/−^ mice die as early as 3 weeks old. Experiments were approved by the Institutional Animal Care and Use Committee of Oakland University and adhered to the Public Health Service Guide for the Care and Use of Laboratory Animals (Department of Health, Education, and Welfare publication, National Institutes of Health 80-23). Blood samples were collected into EDTA-coated Vacutainers and were immediately centrifuged at 3000 rpm for 15 min. The plasma was immediately isolated from the red blood cell pellet and stored in aliquots at −80°C until lipid analysis.

### Liquid chromatography/ mass spectrometry (LC/MS)

Plasma samples were prepared for LC-MS analysis of lipid mediators by the lipidomics Core Facility (Wayne State University, MI) as shown previously [34]. Briefly, samples (0.85 ml) were spiked with 5 ng each (in 150 μl methanol) of 15(S)-HETE-d8,14(15)-EpETrE-d8, Resolvin D2-d5, Leukotriene B4-d4, and Prostaglandin E1-d4 as internal standards for recovery and quantitation and mixed thoroughly. The samples were then extracted for PUFA metabolites using C18 extraction columns as described earlier [34]. LC-MS analysis of the extracted samples for fatty acyl lipid mediators was performed as previously published [35, 36].

### Statistical Analysis

All data are presented as mean ± SEM. Data were analyzed using non-parametric t-test followed to compare the changes in plasma levels of each individual lipid product in cβs^+/−^ vs. WT control mice utilizing Graph Pad Prism Version 4.0 software (Graph Pad Software Inc., La Jolla, CA). For all comparisons, P<0.05 was considered statistically significant (n = 8). To compare the changes in the levels of a group of lipid metabolites derived from specific enzymatic pathway (LOX, COX or CYP) or derived from specific substrate (omega-3, omega-6, AA or LA), we first calculated the fold change in each metabolite in plasma from cβs^+/−^ mice vs. plasma from WT mice followed by non-parametric t-test where *P* < 0.05 was also considered significant. The sample size justification for this study was based on the two-sample t-test. The power of this test depends on (1) the significance level, (2) the anticipated fold change (FC) difference when comparing the two groups of mice (e.g., wild-type vs. those that are deficient in the cβs enzyme), and (3) the true coefficient of variation (CV) of the dependent variable of interest (e.g., level of an individual lipid product). For the t-test, a sample size of n = 8 mice per comparison group would yield at least 80% power to detect the FC values. The CV values included in Table 1 are comparable to those published previously examining the same lipid profiles presented in our study [37].

## Results

### Effect of HHcy on lipoxygenase activity

**Fig. 1:**
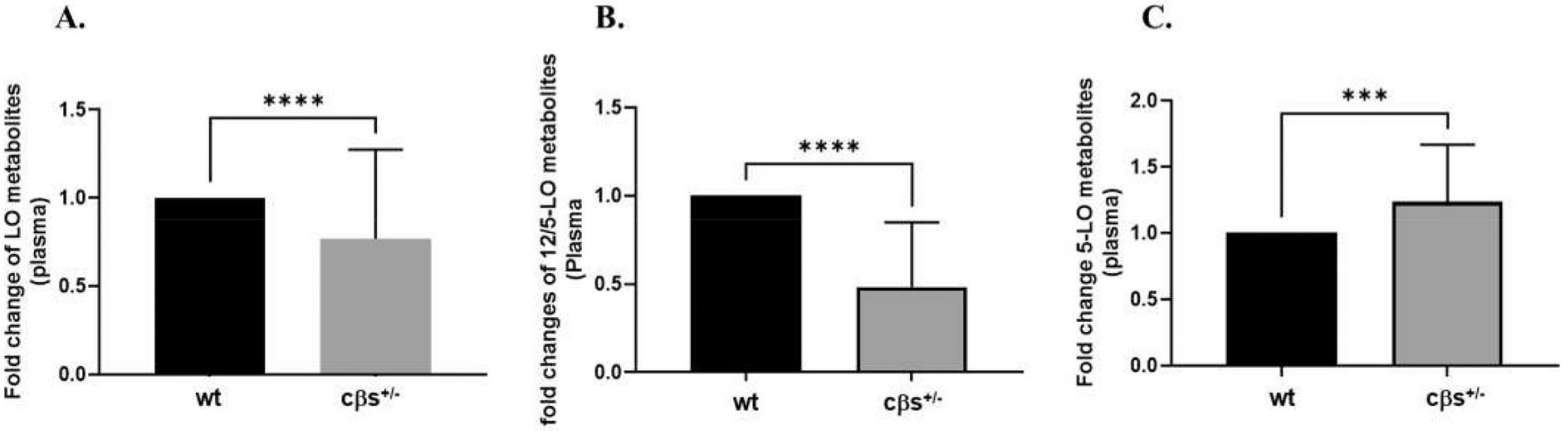
The plasma fold changes of 5-, 12- and 15-LOX metabolites from various substrates including AA, LA, DHA and EPA in cβs^+/−^ vs. WT control mice. There was a significant decrease in total LOX metabolites from all substrates in cβs^+/−^ vs. WT control mice. Although 12/15-LOX metabolites (12- and 15-HETEs) were significantly decreased in cβs^+/−^ vs. WT control, there was no significant difference in the 5-LOX metabolite 5-HETE between cβs^+/−^ and WT control mice (n = 8 per group, *P < 0.05 is considered significant vs. WT control group).

We first evaluated the changes in the levels of individual metabolites of 5-, 12- or 15-LOX derived from various substrates (AA, LA, long-chain PUFA eicosapentaenoic acid (EPA) or docosahexaenoic acid (DHA) (Table 1). There were significant decreases in plasma levels of 12- and 15-HETEs metabolites of AA and the 14- and 17-HDoHE metabolites of DHA in cβs^+/−^ compared to the WT control mice whereas no significant changes were observed in the levels of LA or EPA metabolites. We then compared the fold changes of 5-, 12- and 15-LOX metabolites from various substrates including AA, LA, DHA and EPA in the plasma of cβs^+/−^ vs. WT control mice. This collectively included the fold changes of AA metabolites (5-HETE, 8-HETE, 9-HETE, 11-HETE, 12-HETE, tetramer 12-HETE and 15-HETE), LA metabolites (9-HODE, 13-HODE, 9-OxoODE and 13-OxoODE), EPA metabolites (11-, and 15(S)-HEPEs) and DHA metabolites (4, 7, 11, 14, and 17-HDoHEs). There was a significant reduction in total LOX metabolites from all substrates in cβs^+/−^ vs. WT control mice (Fig. 1A). The fold changes in the metabolites of each individual LOX in cβs^+/−^ vs. WT control mice were also assessed. We noticed a significant reduction (40%) in 12/15-LOX metabolites (12- and 15-HETEs) in cβs^+/−^ vs. WT control mice (*P* < 0.0001, Fig. 1B), however the fold change in the level of 5-HETE, as a reflection of 5-LOX activity, was higher in cβs^+/−^ vs. WT control mice but was not significant (P = 0.07, Fig. 1C).

**Fig. 2:**
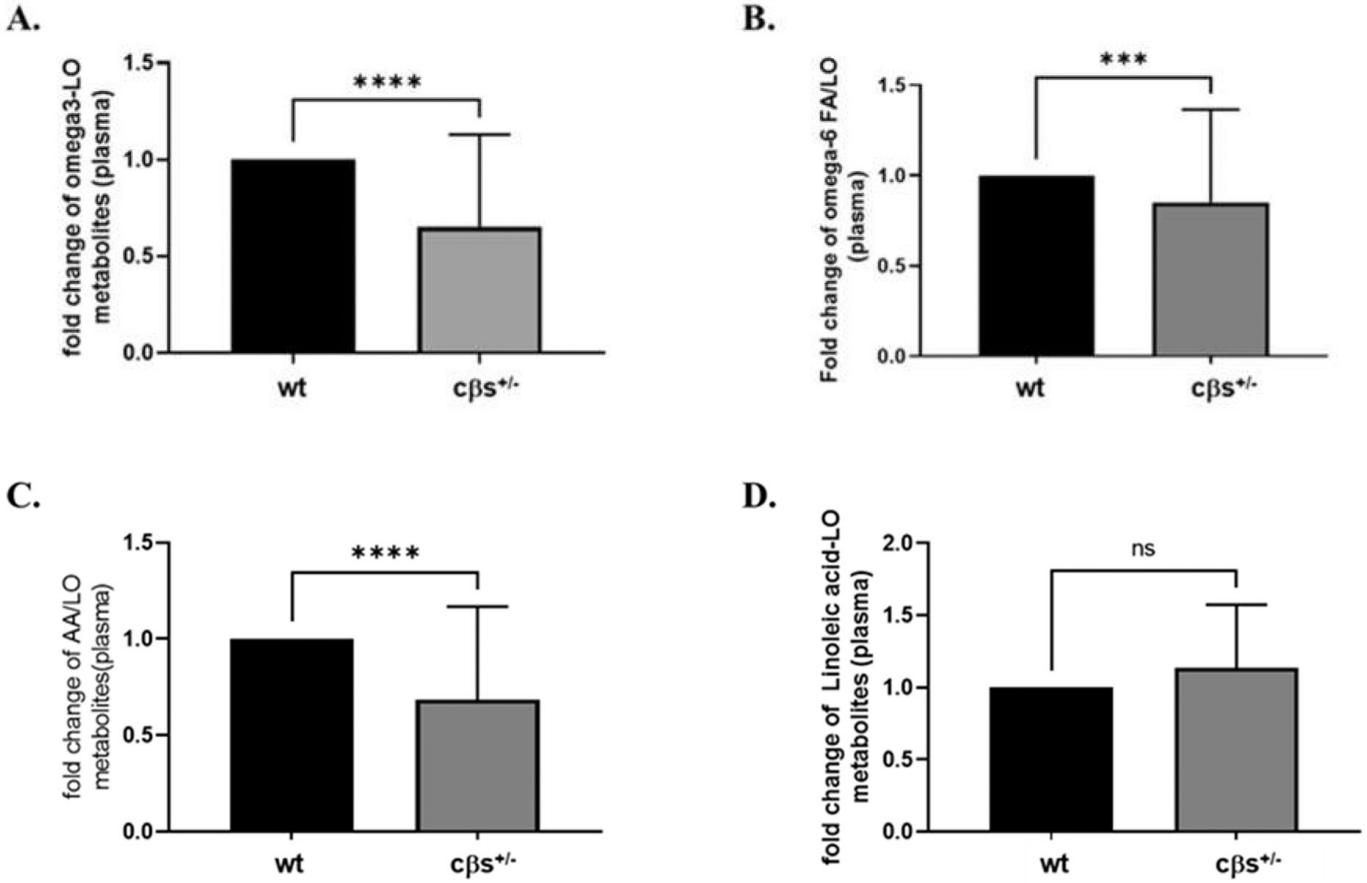
Analysis of the fold changes in plasma LOX-derived metabolites from omega-3, omega-6, AA and LA PUFA in cβs^+/−^ and WT control mice. LOX-derived metabolites from omega-3 and AA PUFA were significantly elevated in cβs^+/−^ vs. WT control mice whereas there was no significant difference in plasma LOX-derived metabolites from omega-6 and LA PUFA between two mice groups (n = 8 per group, *P < 0.05 is considered significant vs. WT control group).

LOX-derived metabolites from omega-3 PUFA (EPA and DHA) as collectively represented by HEPEs and HDoHEs respectively also significantly decreased in cβs^+/−^ mice compared to WT control mice (*P* = 0.008, Fig. 2A). However, analysis of the fold changes of the LOX-derived metabolites from omega-6 PUFA (AA and LA), as represented by HETES and HODEs/OxoODEs respectively, showed no significant difference between cβs^+/−^ and WT control mice (*P* = 0.147, Fig. 2B). We further analyzed the fold changes in LOX-derived metabolites only from AA (5-, 8-, 9-, 11-, 12-, and 15-HETEs) or LA (9- and 13-HODE and 9- and 13-OxoODE) in cβs^+/−^ vs. WT control. Although we noticed a significant reduction in LOX-derived metabolites from AA in cβs^+/−^ vs. WT control mice (*P* = 0.016, Fig. 2C), there was no significant difference in LOX-derived metabolites from LA between the two mice groups (*P* = 0.346, Fig. 2D).

### Effect of HHcy on COX activity

**Fig. 3:**
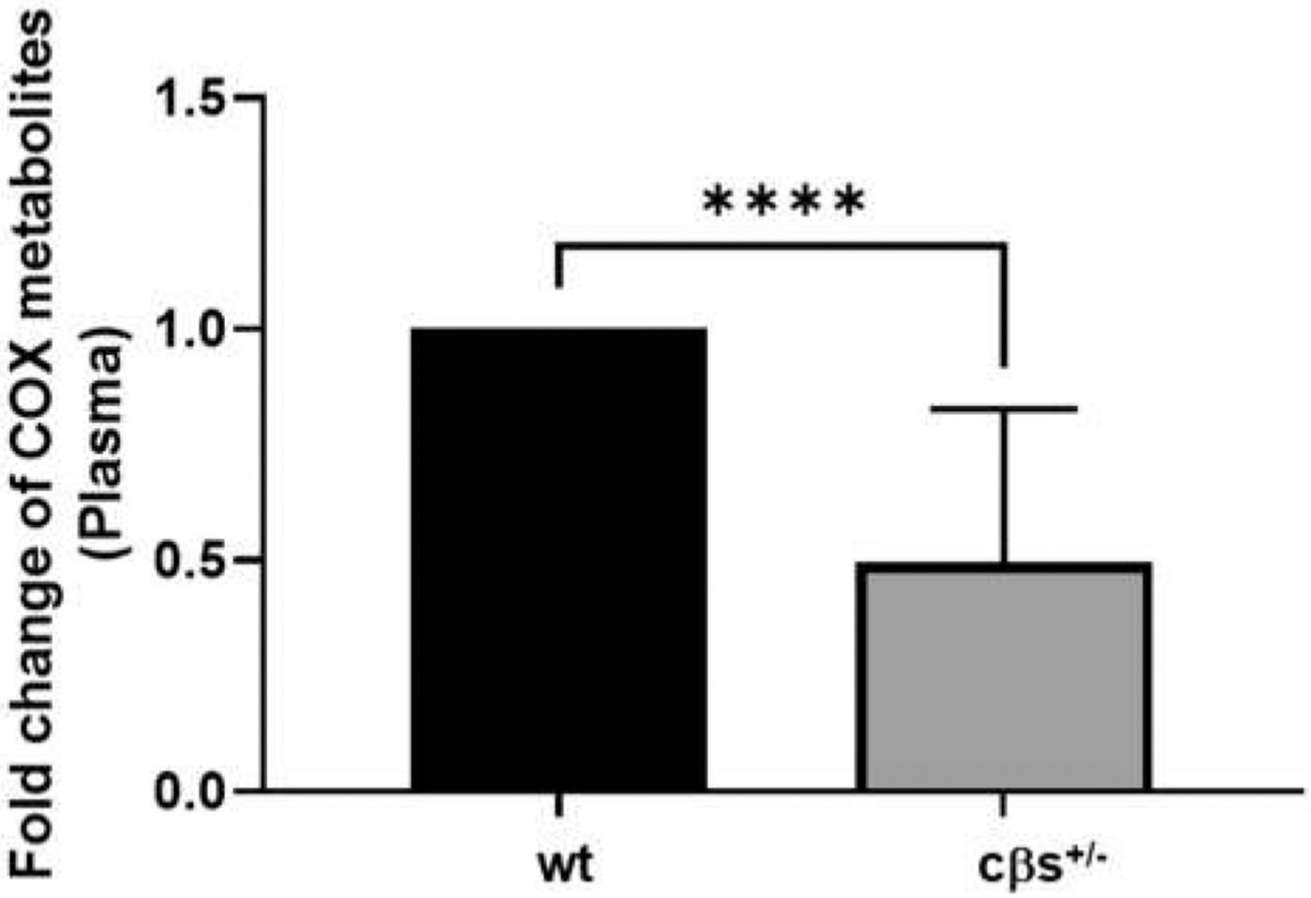
Assessment of the fold changes in plasma COX-derived metabolites levels in cβs^+/−^ and WT control mice. Plasma COX-derived metabolites significantly decreased in cβs^+/−^ compared to WT control mice (n = 8 per group, *P < 0.05 is considered significant vs. WT control group).

We analyzed the fold changes in the levels of COX metabolites both separately and collectively together. Analysis of the fold change in the levels of several COX metabolites collectively (PGE2, PGD2, TXB2, PGA2, PGJ2, 6kPGF1a, PGF2a, 13,14dh-15k-PGD2, D12-PGJ2, 13,14dh-15k-PGD2, 15-keto PGF2a, 19(R)-OH PGF2a & 20-OH PGF2a, and PGD3) showed a significant decrease in the total metabolites in cβs^+/−^ mice in comparison to the control group (*P* = 0.0014, Fig. 3). However, comparing individual COX metabolites separately showed no significant difference between cβs^+/−^ and WT control mice except for the levels of PGD2 which showed significant decrease in cβs^+/−^ mice compared to the WT control mice (Table 2).

### Effect of Hhcy on Cytochrome P450 (CYP)

**Fig. 4:**
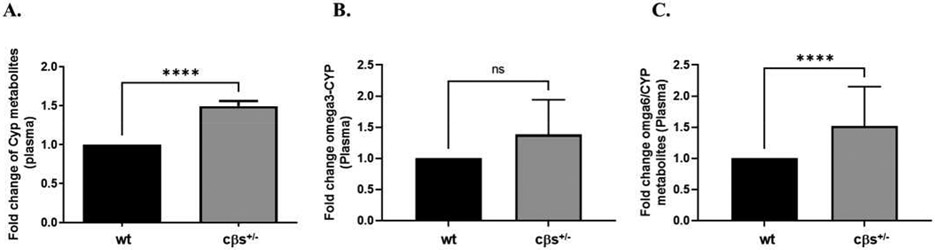
The plasma fold changes in levels of CYP metabolites derived from all substrates (AA, LA and DHA) in cβs^+/−^ vs WT control mice. The plasma fold changes in CYP metabolites were significantly elevated in cβs^+/−^ vs. WT control mice (A). CYP metabolites derived from omega-3 (DHA) showed a modest increase in cβs^+/−^ but was not significant (B). On the other hand, omega-6 metabolites of the AA and LA PUFA were significantly elevated in cβs^+/−^ vs. WT control mice (C) (n = 8 per group, *P < 0.05 is considered significant vs. WT control group).

**Fig. 5:**
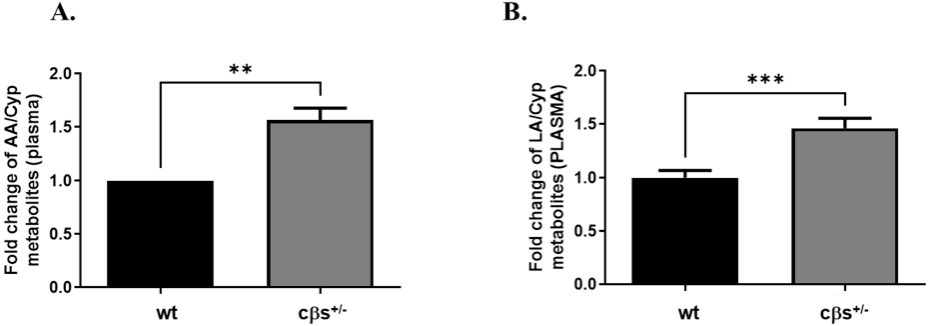
Assessment of the fold changes in the plasma CYP-derived metabolites from AA (A) and LA (B) in cβs^+/−^ vs. the control WT mice. The fold changes in plasma CYP-derived metabolites from AA and LA were significantly elevated in cβs^+/−^ vs. WT control mice (n = 8 per group, *P < 0.05 is considered significant vs. WT control group).

Analysis of individual metabolites of CYP showed significant increases in the levels of 20HETE, 5(6)-EpETrE (derivatives of AA) and the 12, 13-DiHOME (a derivative of LA) in cβs^+/−^ compared to WT control mice (Table 3). Moreover, the EpETre/DiHETrE ratio was significantly decreased in cβs^+/−^ compared to WT mice (Table 3). We then evaluated the fold changes in plasma levels of CYP metabolites derived from all substrates (AA, LA and DHA) in cβs^+/−^ vs. WT control mice. This included the fold changes in AA metabolites (5(6)-, 11(12) - and 14(15) - EpETrEs and their corresponding DiHETrEs), LA metabolites (9(10) - and 12(13) - EpOMEs and DiHOMEs) and DHA metabolites (19, 20- DiHDoPE and 7(8) - and 10(11)-EpDPE). Plasma CYP metabolites derived from all substrates significantly increased in cβs^+/−^ compared to the WT control mice (*P* < 0.0001, Fig. 4A). CYP-metabolites derived from omega 3 (DHA) showed a modest increase in cβs^+/−^ but was not significant (Fig. 4B), while metabolites of omega 6 PUFA (both AA and LA metabolites) were significantly elevated in cβs^+/−^ vs. WT control mice (Fig. 4C). Finally, we analyzed the fold change in the plasma levels of CYP-derived metabolites from each individual substrate separately. Plasma AA- and LA-derived metabolites are significantly increased in cβs^+/−^ compared to the WT control mice (Fig. 5A and 5B, respectively).

### HHcy activates sEH

**Fig. 6:**
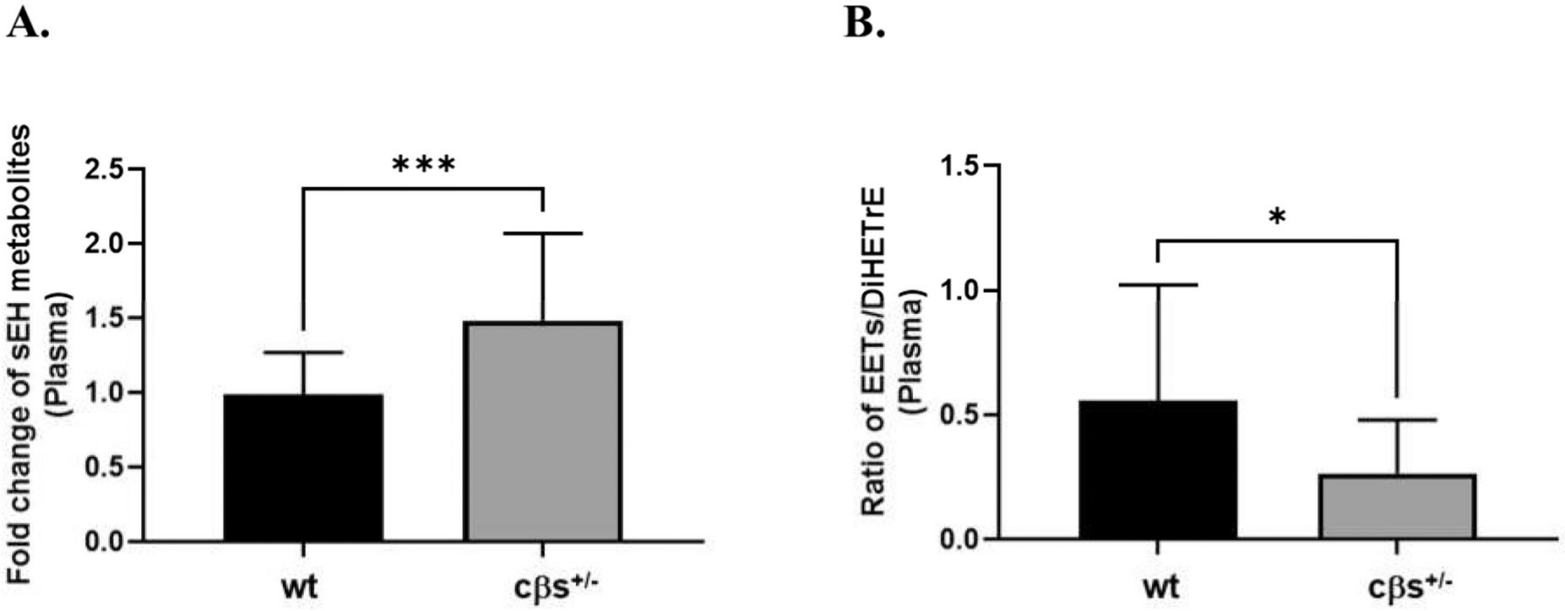
Fold change of plasma sEH metabolites in cβs^+/−^ mice relative to the control WT group. There was a significant increase in sEH metabolites in cβs^+/−^ compared to the WT control mice (A). Plasma EETs and DiHETrEs ratio, an indicative of EETs bio-availability (B), also significantly decreased in cβs^+/−^ compared to the WT control mice (n = 8 per group, *P < 0.05 is considered significant vs. WT control group).

Although EETs are AA metabolites with anti-inflammatory and vascular protective effects, their physiological relevance is limited by their rapid degradation by sEH to less active DHETrEs. To evaluate the impact of HHcy on sEH activity, we assessed the fold changes in sEH metabolites from various substrates (AA, LA or DHA) such as DiHETrEs, DiHOME or DiHDOPE, respectively in cβs^+/−^ vs. WT control mice. There was a significant increase in sEH metabolites in cβs^+/−^ vs. WT control mice (Fig. 6A). The increase in sEH activity in cβs^+/−^ vs. the WT mice was associated with decreased EETs availability as shown by a significant decrease in the plasma EETs/DHETrEs ratio in cβs^+/−^ mice compared to WT control. This ratio represented the ratio of the fold changes in the plasma levels of 5(6),11, 12 and 14, 15 EET to their corresponding DiHETrEs metabolites (Fig. 6B). These data suggest that increased sEH activity in HHcy mice could limit the vascular protective effect of EETs via increasing EETs metabolism and lowering EETs bio-availabilities.

## Discussion

The role of Hhcy in the development of CVD has been well established [1]. However, the underlying mechanisms remain to be further explored. Previous studies suggest that elevation in oxidative stress and inflammatory cytokines together with decreased nitric oxide bio-availability are contributing factors to the incidence and progression of CVD complications of HHcy [8, 10, 12]. Recent studies have explored the role of bioactive lipids as potential mediators for HHcy-induced CVD complications [13, 25]. Our current study underscores the changes in the metabolism of bioactive lipids (eicosanoids) derived from PUFA (AA, LA, EPA and DHA) as potential downstream mediators to HHcy. Utilizing cystathionine-β-synthase heterozygous (cβs^+/−^) mice as a model of HHcy, our data revealed that plasma levels of LOX and COX metabolites decreased whereas CYP metabolites significantly increased in cβs^+/−^ vs. WT control mice. Our findings suggest that dysregulation of eicosanoid production could be a contributing factor to the systemic complications of HHcy in CVD.

HHcy is associated with enhanced peroxidation of AA to form bioactive F (2)-isoprostane, a marker of oxidative stress, linking HHcy to platelet activation/aggregation in cystathionine-β synthase deficiency patients [38]. HHcy is also linked to the development of diabetic retinopathy (DR) and age-related macular degeneration (AMD) [39, 40]. Our group has previously shown that NFκB inflammatory signaling activation, downregulation of anti-inflammatory cytokines and elevation in oxidative stress are key mediators in retinal vascular dysfunction associated with HHcy [41-43]. Similarly, our group has established the role of bioactive lipids derived from AA metabolism by 12/15-LOX and CYP in the development of retinal microvascular dysfunction in experimental diabetes via enhancing oxidative stress and inflammatory signaling [21, 39, 44-46]. Furthermore, the role of HHcy on renal injury/failure has been established and we have previously shown a correlation between dysregulation of AA metabolism by 12/15-LOX, COX or CYP and the pathogenesis of diabetic and acute renal injury [29, 34]. In general, several studies have shown that altered levels of Hcy, through AA release and metabolism, can influence the synthesis and the activity of PGs, PGI2, TXA, EETs, and HETEs [47]. Accordingly, we developed special interest in investigating whether HHcy impact LOX, COX and CYP activities and subsequently the levels of circulating bioactive lipids.

Accumulating evidences demonstrated that homocysteine upregulates brain 5-LOX to produce 5-HETE and deletion of 5-LOX attenuated neurodegeneration and amyloid β formation which are relevant to the pathogenesis of Alzheimer’s disease [48-50]. There was no data in the literatures linking homocysteine and overall LOX activities. Our data showed no significant changes in plasma levels of 5-HETE in cβs^+/−^ vs. control mice suggesting that local brain changes in 5-LOX expression and activity could be more important than the circulating levels in mediating brain structural and functional changes in HHcy.

Omega-3 fatty acids EPA and DHA are metabolized by LOX producing resolvins, which promote anti-atherogenic signaling through the stimulation of endogenous resolution of inflammation [51, 52]. In our study, we noticed that HHcy decreased overall LOX as well as 12/15-LOX activities as shown by decreased 12- and 15-HETEs production contrary to what we did expect since 12- and 15-HETEs are known to have proinflammatory properties. However, the decrease in LOX metabolites was also associated with a marked decrease in both omega-3 and omega-6 metabolites. Whether dysregulation of LOX activity/metabolites plays a role in CVD complications of HHcy will remain to be further investigated.

COX derived metabolites contribute to the regulation of vascular tone and platelet aggregation. For example, TXA2 is a powerful platelet aggregating factor and vasoconstrictor that is mainly derived from COX-1 while prostacyclin is a potent anti-aggregating agent and vasodilator [53, 54]. Homocysteine is known to increase AA metabolites PGD2 and TXB2 by upregulating the expression of the COX enzymes, which might contribute to the endothelial cell activation and platelet aggregation [47]. However, previous findings also suggest that homocysteine Inhibited COX activity in human endothelial cells [55]. In addition, recent studies revealed a novel role for COX metabolites in the pathogenesis of homocysteine-induced proinflammatory response in neurons [56]. In our current study, HHcy decreased COX-derived AA metabolism including vasoconstrictor and vasodilator metabolites. The metabolism of omega-3 PUFA by COX and LOX enzymes not only generates 3-series prostaglandins and leukotrienes but also unique omega-3 autacoids such as resolvins and protectins, which have anti-inflammatory or anti-angiogenic effects. Thus, COX-derived alteration in omega-3 PUFA metabolism in cβs^+/−^ mice vs. WT control could drive vascular injury via decreased anti-inflammatory metabolites levels.

Besides the extensively studied COX and LOX pathways, omega-3 and omega-6 PUFA are also substrates of CYP epoxygenases, which convert them to epoxy signaling lipids including EETs derived from omega-6 AA and epoxydocosapentaenoic acids (EDPs) from omega-3 DHA [57-59]. Analysis of plasma omega-3 and omega-6 metabolites of each of the LOX, COX and CYP enzymatic pathways demonstrated changes in their plasma total metabolites in cβs^+/−^ vs. WT control mice. Our data clearly demonstrated significant reductions of plasma omeg-3 and omega-6 metabolites of LOX and COX pathways while there was significant increase in both plasma omega-3 and omega-6 metabolites of CYP pathway. DHA, the most abundant omega-3 PUFA in most tissues, can efficiently compete with AA for CYP epoxygenases metabolism, leading to replacement of EETs with EDPs in vivo [57-59]. EETs and EDPs are autocrine and paracrine mediators to regulate inflammation and vascular tone, however EDPs have been reported to have more potency on vasodilation and anti-inflammation than EETs [57-59].

Since CYP metabolites are known to play a crucial role in the pathogenesis of vascular injury, our data clearly showed significant elevations in CYP metabolites in cβs^+/−^ vs. WT control mice. We also found significant increases in plasma levels of omega-3 and omega-6 metabolites in cβs^+/−^ mice. In particular, the level of 20-HETE, which is known to have inflammatory, fibrotic and vasoconstrictor properties [60], is significantly elevated in cβs^+/−^ vs. WT control mice. Beside the change in hydroxylase metabolite 20-HETE, our data revealed a significant reduction in the availability of the anti-inflammatory epoxygenase metabolites EETs, as evident by decreasing EETs/DiHETrEs ratio, in cβs^+/−^ compared to WT control mice suggesting increased activity of the EETs metabolizing enzyme sEH. Increased activity of sEH is associated with several cardiovascular complications via enhancing EETs degradation to less active DHETrEs and subsequently decrease EETs availability [28, 29]. HHcy is associated with atherosclerotic events involving the modulation of AA metabolism and the activation of matrix metalloproteinase-9 (MMP9) [61]. CYP epoxygenase-2J2 (CYP2J2) is abundant in the heart endothelium, and its AA metabolites EETs mitigates inflammation through NF B-induced MMP-9 activation. Moreover, epoxygenase transfection or exogenous addition of 8, 9-EET attenuated homocysteine-induced NF B activation and subsequently inhibited MMP-9 activity [61]. Consistent with these findings, our data suggest that increased sEH activity and decreased EETs bio-availability in cβs^+/−^ mice could be a contributing factor to HHcy-induced vascular injury.

It is not clear how HHcy increases sEH activity to limit EETs bio-availability. Previous studies have shown that homocysteine-induced sEH upregulation is associated with activation of activating transcription factor-6 (ATF6) [16]. Bioinformatics analysis also revealed a putative ATF6-binding motif in the promoter region of the sEH gene. Homocysteine treatment or ATF6 overexpression promoted ATF6 binding to the sEH promoter and increased its activity [16]. Thus, the authors conclude that ATF6 activation and DNA demethylation may coordinately contribute to Hcy-induced sEH expression and endothelial activation [16]. Based on our findings that sEH activity increases to limit EETs availability in HHcy, we will utilize the physiological approach as our future direction to determine whether sEH inhibition or stable EETs analogs might be a new therapeutic approach for alleviating HHcy -induced vascular injury in CVD.

In conclusion, HHcy is associated with dysregulation of the circulating PUFA-derived eicosanoids as shown by a significant increase in CYP-derived metabolites and decreased both LOX- and COX-derived metabolites in addition to decreased plasma EETs availability. These changes in circulating eicosanoids suggest that the PUFA metabolism could be a downstream signal in HHcy to mediate vascular injury in CVD. Further studies are needed to dissect the role of each enzymatic pathway and the molecular mechanisms behind these changes during HHcy.

## Figures and Tables

**Table 1.** Effect of hyperhomocysteinemia on plasma levels of lipoxygenase lipid metabolites.

Lipid	Plasma (ng/ml)		
	Wild type	cβs^+/−^	
	Mean ± SE	Mean ± SE	*P* value
1- Lipoxygenase products			
A) AA metabolites			
- 12-HETE	43.07 ± 16.06	3.81 ± 1.74	0.02*
- 15-HETE	1.29 ± 0.25	0.67 ± 0.13	0.04*
- 9-HETE	0.18 ± 0.02	0.18 ± 0.02	0.96
- 8- HETE	0.92 ± 0.10	0.73 ± 0.12	0.26
- 5-HETE	4.06 ± 0.54	4.40 ± 0.37	0.60
- 11-HETE	2.49 ± 0.65	1.60 ± 0.29	0.23
B) EPA metabolites			
- 12-HEPE	1.90 ± 0.80	0.17 ± 0.10	0.05
- 15-S-HEPE	0.13 ± 0.02	0.06 ± 0.01	0.05
- 11-HEPE	0.06 ± 0.01	0.05 ± 0.01	0.75
C) LA metabolites			
- 13-HODE	23.68 ± 2.96	31.45 ± 4.32	0.15
- 9-HODE	3.02 ± 0.38	4.09 ± 0.53	0.12
- 9-OxoODE	2.46 ± 0.31	2.68 ± 0.24	0.59
-13-OxoODE	2.85 ± 0.33	2.25 ± 0.18	0.14
D) DHA metabolites			
-4-HDoHE	1.42 ± 0.25	1.57 ± 0.16	0.51
-7-HDoHE	0.20 ± 0.02	022 ± 0.03	0.63
- 11-HDoHE	0.36 ± 0.04	0.42 ± 0.06	0.57
- 14-HDoHE	2.60 ± 0.68	0.81 ± 0.20	0.03*
- 17-HDoHE	0.40 ± 0.09	0.17 ± 0.03	0.01*

* *P* < 0.05

**Table 2.** Effect of hyperhomocysteinemia on Plasma levels of Cyclogeneses-derived metabolites

Lipid	Plasma (ng/ml)		
	Wild type	cβs^+/−^	
	Mean ± SE	Mean ± SE	*P* value
3. Cycloxygenase			
- PGA2	0.05 ± 0.01	0.14 ± 0.09	0.27
- PGD2	0.10 ± 0.02	0.05 ± 0.01	0.04*
- PGE2	0.31 ± 0.12	0.10 ± 0.02	0.10
- PGJ2	0.03 ± 0.00	0.02 ± 0.00	0.24
- TXB2	0.57 ± 0.30	0.26 ± 0.17	0.49
- 13-HDoHE	0.36 ± 0.04	0.37 ± 0.06	0.80
- 13,14dh-15k-PGD2	0.19 ± 0.06	0.08 ± 0.01	0.07
- 19(R)-OH PGF2a & 20-OH PGF2a	0.03 ± 0.00	0.04 ± 0.01	0.41
- 6kPGF1a	0.32 ± 0.13	0.13 ± 0.05	0.25

* *P* < 0.05

**Table 3.** Effect of hyperhomocysteinemia on Plasma levels of CYP-induced metabolites

Lipid	Plasma (ng/ml)		
	Wild type	cβs^+/−^	
	Mean ± SE	Mean ± SE	*P* value
2. Cytochrom-P450			
A) AA metabolites			
- 20-HETE	0.56 ± 0.11	0.94 ± 0.12	0.04*
- 5(6)-EpETrE	0.05 ± 0.01	0.08 ± 0.01	0.01*
- 11(12)-EpETrE	0.12 ± 0.01	0.15 ± 0.02	0.27
- 14,15-EpETrE	0.13 ± 0.04	0.10 ± 0.01	0.80
- 5(6)DiHETrE	0.15 ± 0.02	0.18 ± 0.02	0.33
- 11,12-DiHETrE	0.15 ± 0.03	0.21 ± 0.04	0.18
- 14,15-DiHETrE	0.73 ± 0.17	1.13 ± 0.18	0.12
- EpETre/DiHETrE ratio	0.56 ± 0.09	0.27 ± 0.04	0.02*
B) LA metabolites			
- 9(10)-EpOME	1.60 ± 0.14	1.96 ± 0.24	0.22
- 12(13)-EpOME	1.10 ± 0.15	1.65 ± 0.22	0.06
- 9,10-DiHOME	1.29 ± 0.22	1.97 ± 0.26	0.06
- 12,13-DiHOME	3.91 ± 0.65	6.25 ± 0.78	0.03*
C) DHA metabolites			
- 19,20 DiHDoPE	0.80 ± 0.10	1.03 ± 0.10	0.16
- 7,8 EpDPE	0.03 ± 0.00	0.06 ± 0.01	0.11
- 10,11 EpDPE	0.09 ± 0.01	0.13 ± 0.02	0.10

* *P* < 0.05

## Data Availability

Please contact the corresponding author for detailed data in this article.
